# Efficacy of an antimicrobial stewardship intervention for early adaptation of antibiotic therapy in high-risk neutropenic patients

**DOI:** 10.1186/s13756-023-01354-5

**Published:** 2024-01-17

**Authors:** Claire Durand, Karine Risso, Michael Loschi, Nicolas Retur, Audrey Emery, Johan Courjon, Thomas Cluzeau, Michel Carles

**Affiliations:** 1 Infectious Disease Department, University Hospital of Nice, Cote D’Azur University, Nice, France; 2 Hematology Department, University Hospital of Nice, Cote D’Azur University, Nice, France; 3 Université Côte d’Azur, INSERM U1065, Cote D’Azur University, Nice, C3M France; 4 Pharmacy Department, University Hospital of Nice, Cote D’Azur University, Nice, France; 5 Bacteriology Department, University Hospital of Nice, Cote D’Azur University, Nice, France

**Keywords:** Antibiotic, Antimicrobial stewardship, Hematology, Febrile neutropenia

## Abstract

**Background:**

The 4th European Conference on Infections in Leukemia recommends early adaptation of empirical antibiotic therapy (EAT) for febrile neutropenia in stable patients.

**Objectives:**

To assess the efficacy of an antimicrobial stewardship (AMS) intervention promoting early de-escalation and discontinuation of EAT in high-risk neutropenic patients.

**Methods:**

This before-after study was conducted in the hematology department of the University Hospital of Nice, France. The AMS intervention included the development of clinical decision support algorithms, a twice-weekly face-to-face review of all antibiotic prescriptions and monthly feedback on the intervention. The primary endpoint was overall antibiotic consumption during hospital stay, expressed as days of therapy (DOT).

**Results:**

A total of 113 admissions were included: 56 during the pre-intervention period and 57 during the intervention period. Induction chemotherapy and conditioning for allogeneic stem cell transplantation were the most frequent reasons for admission. In the intervention period, there was a significant decrease in overall antibiotic consumption (median DOT 20 vs. 28 days, *p* = 0.006), carbapenem consumption (median DOT 5.5 vs. 9 days, *p* = 0.017) and anti-resistant Gram-positive agents consumption (median DOT 8 vs. 11.5 days, *p* = 0.017). We found no statistical difference in the rates of intensive care unit admission (9% in each period) and 30-day mortality (5% vs. 0%, *p* = 0.243). Compliance with de-escalation and discontinuation strategies was significantly higher in the intervention period (77% vs. 8%, *p* < 0.001).

**Conclusion:**

A multifaceted AMS intervention led to high compliance with early de-escalation and discontinuation of EAT and lower overall antibiotic consumption, without negatively affecting clinical outcomes.

**Supplementary Information:**

The online version contains supplementary material available at 10.1186/s13756-023-01354-5.

## Introduction

Febrile neutropenia (FN) is a frequent and serious complication in patients with hematological malignancies undergoing intensive chemotherapy [1]. FN episodes are responsible for repeated and prolonged antibiotic therapy, leading to an increased risk of antibiotic resistance [2], *Clostridioides difficile* infections [3], fungal infections [4] and adverse drug events [5]. The spread of antibiotic resistance is a major threat in high-risk neutropenic patients given that a delay in introduction of appropriate empirical antibiotic therapy (EAT) in this population is associated with increased morbidity and mortality [6].

Excessive and inappropriate antibiotic use are major drivers of the emergence and spread of antibiotic resistance [7, 8]. Antimicrobial stewardship (AMS) interventions have therefore been introduced to optimize antibiotic use, in order to decrease unintended consequences of antibiotic use, such as growing antibiotic resistance and excessive healthcare costs [9, 10]. Given the threat of antibiotic resistance and the extensive use of antibiotics in high-risk neutropenic patients, there is an urgent need to implement AMS interventions in hematology departments.

In 2013, the 4th European Conference on Infections in Leukemia (ECIL) group published new guidelines for the management of FN, prompting early adaptation of EAT in stable afebrile patients, regardless of neutrophil count and expected duration of neutropenia [11]. Despite these evidence-based guidelines, de-escalation and discontinuation strategies are not widely implemented in hematology departments [12]. Recent studies have found that early adaptation of EAT is feasible and safe and could lead to reduced antibiotic consumption [13–23]. However, most of these studies solely investigated the effects of one form of adaptation (i.e. de-escalation or discontinuation) [13, 15, 16, 18–21] or focused on specific presentations of FN [13, 15, 18, 19, 21, 24, 25] or patient profiles [14–16, 18, 24]. One randomized controlled trial has found that discontinuation of EAT after 72 h of apyrexia and clinical recovery in high-risk neutropenic patients without microbiological documentation is safe and can significantly reduce unnecessary exposure to antimicrobials [13]. Several interrupted time series studies have demonstrated a significant reduction in carbapenem consumption following implementation of early adaptation of EAT but not a significant reduction in total antibiotic consumption [17, 20, 23].

In response to growing antibiotic resistance and low compliance with ECIL guidelines in the hematology department in our center, we have developed and implemented a multifaceted AMS intervention. This intervention aimed to improve the quality of FN management and to promote the adoption of early de-escalation and discontinuation strategies in high-risk neutropenic patients by the hematology team.

The goal of this before-after study was to assess the impact of a multifaceted AMS intervention on antibiotic consumption and clinical outcomes in high-risk neutropenic patients. Moreover, we sought to assess the applicability of de-escalation and discontinuation strategies as well as the compliance of the hematology team with these strategies.

## Methods

### Study population

This study was conducted in the hematology department of the 1800-bed University Hospital of Nice, France for two 6-month periods (October 2019–March 2020 and December 2021–May 2022). The hematology department is divided into two separate units: an 8-bed hematologic intensive care unit with laminar air flow rooms and a 16-bed conventional hospitalization unit.

During these periods, all consecutive admissions to the hematology department for intensive chemotherapy, with chemotherapy-induced neutropenia lasting seven days or more, and occurrence of at least one febrile episode, were eligible for inclusion. Admissions were excluded if patients were younger than 18 years old, had chemotherapy-induced neutropenia for less than seven days or received corticosteroids.

### Definitions

High-risk neutropenia was defined as neutropenia lasting seven days or more. Fever recurrence was defined as relapse of fever during neutropenia in patients who had been afebrile for at least 48 h. FN episodes were classified as fever of unknown origin (FUO), clinically documented infection (CDI) or microbiologically documented infection (MDI), according to ECIL guidelines [11].

Discontinuation was defined as the cessation of all antibiotic therapy. De-escalation was defined as either switching to a narrower-spectrum beta-lactam according to the consensual ranking by Weiss et al. [26] or stopping one antibiotic of a combination therapy (except for the discontinuation of aminoglycosides after a single injection). Escalation was defined as either switching to a broader-spectrum beta-lactam or adding an antibiotic from a different class.

### Design and implementation of the AMS intervention

The pre-intervention period lasted six months and spanned from October 2019 to March 2020. A pre-intervention period prior to the COVID-19 pandemic was chosen due to a decrease of the AMS team activity during the first wave of the pandemic, which may have impacted antibiotic prescribing practices in the hematology department. During the pre-intervention period, a dedicated hotline for antimicrobial prescribing guidance was provided but there was no systematic review of all antibiotic prescriptions. During that period, early discontinuation of EAT was not performed, while early de-escalation was rarely performed. Laboratory surveillance of antimicrobial resistance among Gram-negative bacteria isolated from blood cultures in the hematology department between January 2018 and December 2020 showed that out of the 29 *Pseudomonas aeruginosa* isolates, 17.2% were resistant to imipenem, 10.3% were resistant to piperacillin-tazobactam and 6.9% were resistant to cefepime. Out of the 141 *Enterobacterales* isolates, 7.8% produced extended spectrum beta-lactamase and 4.3% produced high level cephalosporinase, while 2.8% produced carbapenemase. As for Gram-positive bacteria, out of the 11 *Staphylococcus aureus* and 130 coagulase-negative staphylococci isolates, 9.1% and 70.8% were respectively resistant to methicillin. No vancomycin-resistant enterococci were isolated among the 37 isolates of enterococci.

In early 2021, we designed a persuasive multifaceted AMS intervention, which we implemented in November 2021. This intervention included the development of new local clinical guidelines in the form of visual decision algorithms. These decision algorithms were subsequently discussed and reviewed with the hematology medical team until consensus was reached. These visual decision aids were then displayed in all medical offices of the hematology department. Educational meetings were held with medical residents and paramedical staff during the implementation phase. Additionally, a patient-level review of all antibiotic prescriptions was conducted twice a week for a period of six months in the presence of the hematology medical team and the AMS team. Patient-specific recommendations regarding antibiotic therapy were provided by the AMS team based on clinical presentation and results of microbiological samples. Moreover, the AMS team offered day-to-day guidance via a dedicated hotline.

The intervention phase started in December 2021 and lasted for the subsequent six months, until May 2022. During that time, antibiotic consumption, clinical outcomes and compliance with ECIL guidelines were prospectively assessed and face-to-face meetings were organized monthly by the AMS team to provide feedback on the progress of the intervention and to discuss opportunities for improvement with the hematology team.

### Febrile neutropenia guidelines

**Fig. 1:**
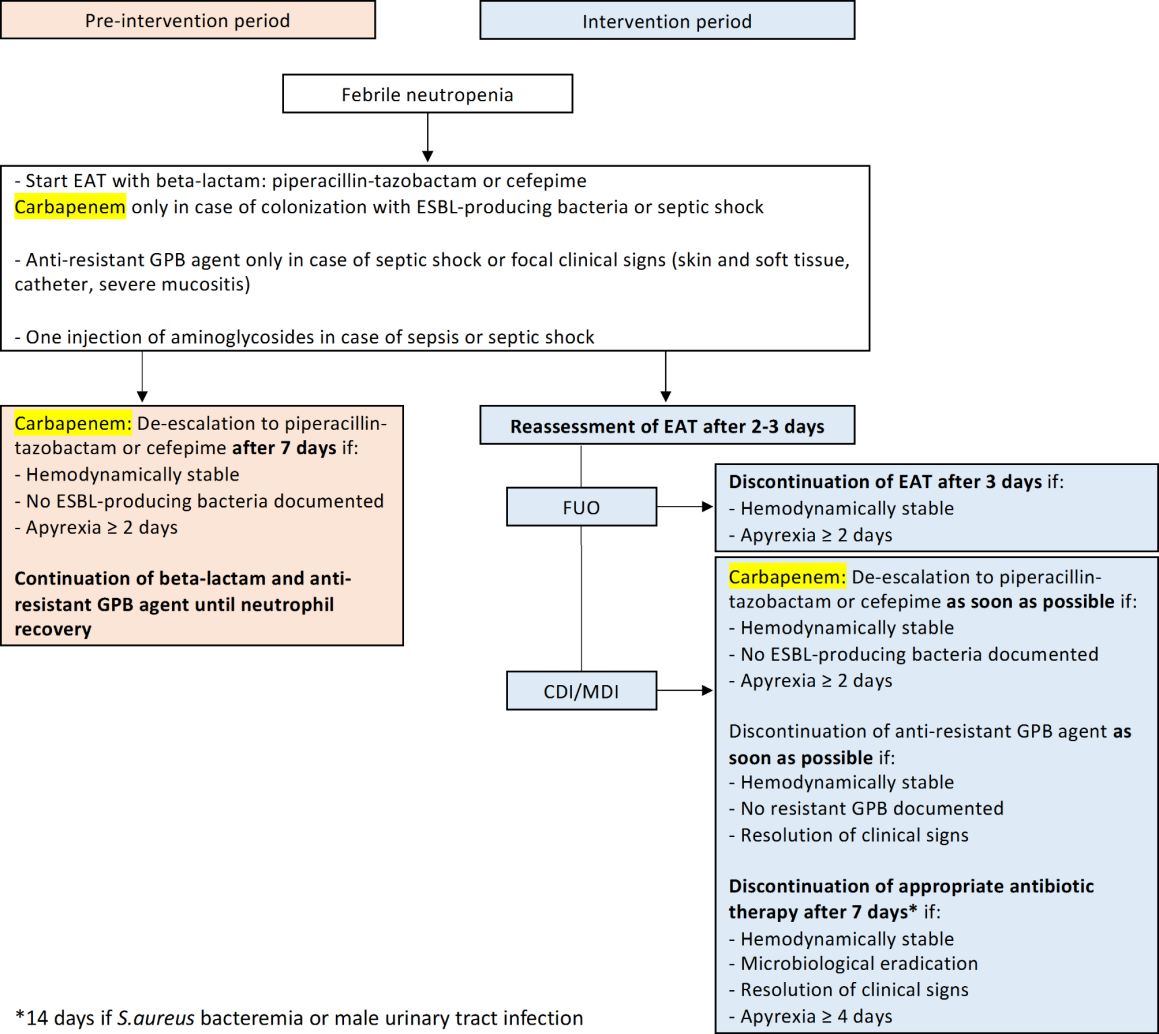
Summary of febrile neutropenia guidelines during the pre-intervention and the intervention periods. *Abbreviations*: EAT: Empirical antibiotic therapy; ESBL: Extended-spectrum beta-lactamase; GPB: Gram-positive bacteria

During the intervention period, EAT was systematically reassessed between 48 and 72 h after introduction. Early adaptation of EAT was encouraged in stable patients according to ECIL criteria, as shown in Fig. 1. If fever persisted in stable patients without new clinical signs, changes in antibiotic therapy were discouraged and the diagnostic work-up was continued. If fever recurred after antibiotic discontinuation, EAT was immediately reintroduced, using the same class of antibiotics as before, after new blood cultures were taken. Detailed FN guidelines and decision algorithms are available in the Supplementary material (Figure S1 to S3).

Neither gut decontamination nor bacterial prophylaxis were used during the study. Antifungal prophylaxis was used according to ECIL guidelines.

### Data collection

Individual data on the pre-intervention period were retrospectively collected through a review of electronic health records (EHRs) while data on the intervention period were prospectively collected. All data on antibiotic consumption were retrieved from EHRs and were then double checked for accuracy by the pharmacy department. The applicability of de-escalation and discontinuation strategies and clinician compliance with these strategies were assessed for all FN episodes by the AMS team. Clinician compliance was defined as the application of de-escalation or discontinuation of EAT within 48 h of applicability according to the algorithms.

The primary endpoint was overall antibiotic consumption during hospital stay, expressed as days of therapy (DOT). All doses of a specific antibiotic administered on a given day were counted as one DOT [27]. The DOT for a given stay was the sum of DOT for each antibiotic [27]. Secondary endpoints included length of therapy (LOT), antibiotic-free days (AFD), 30-day mortality, intensive care unit (ICU) admission, *C. difficile* infection and length of stay. LOT was defined as the number of days that a patient received antibiotic therapy, irrespective of the number of drugs [27]. AFD were defined as the number of days that a patient did not receive antibiotic therapy between the onset of the first FN episode and neutrophil recovery. We defined 30-day mortality as death occurring within 30 days of the onset of neutropenia.

### Statistical analysis

Continuous variables are reported as median (interquartile range) and categorical variables as number (percentage). Continuous variables were compared using the Student’s t-test or the Mann-Whitney test, when appropriate. Categorical variables were compared using the Chi-2 test or the Fisher’s exact test, when appropriate. All tests were two-tailed and p-values ≤ 0.05 were considered statistically significant. Univariate and multivariate logistic regression was performed to identify factors associated with clinician compliance with early de-escalation and discontinuation strategies. Only variables with p-values < 0.1 in the univariate analysis were included in the multivariate logistic regression model. Sample size was calculated using a power of 80% and an alpha value of 0.05. Based on preliminary data from our hematology department, mean DOT per stay was 29 days (standard deviation = 13.5 days) and a reduction of DOT by 25% in the intervention period was considered significant. Based on these assumptions, a sample size of 55 per period was required. The open-source software R Foundation for Statistical Computing (Vienna, Austria) was used for statistical analysis.

## Results

A total of 113 admissions were included, according to the inclusion criteria: 56 during the pre-intervention period and 57 during the intervention period, involving 47 and 48 patients respectively.

Four educational meetings were held with medical residents and paramedical staff during the implementation phase. Forty reviews of antibiotic prescriptions were carried out by the AMS team during the implementation and the intervention phases, while 4 face-to-face meetings were held with the hematology medical team during the intervention phase to provide feedback on the intervention.

### Sample characteristics

Patient and admission characteristics were similar between the two periods, as displayed in Table 1. FN episodes characteristics are summarized in Table 2. Patients experienced more febrile episodes per stay during the intervention period, although this difference was not statistically significant. The median time between the first episode of FN and neutrophil recovery was 19 days in both periods. Overall, distribution of etiologies of FN episodes did not differ between the two periods. Bacterial species and their antibiotic susceptibility isolated from blood cultures in bacteremia during the two periods are described in the Supplementary material (Table S1). Frequency of fever recurrence and time to recurrence were also similar during the two periods. Moreover, there was no statistical difference in the occurrence of sepsis and septic shock between the two periods.

**Table 1:** Main characteristics of patients and admissions

Patient characteristics	Pre-intervention period (n = 47)	Intervention period (n = 48)	*p* value
Age (years)	61 (44.5–66)	60.5 (54–67.25)	0.099
Sex (female), n (%)	26 (55)	18 (38)	0.082
Underlying hematologic disease, n (%)			0.065
Acute myeloid leukemia	22 (47)	29 (61)	
Acute lymphoblastic leukemia	3 (6)	0 (0)	
Multiple myeloma	7 (15)	5 (10)	
Lymphoma	9 (20)	2 (4)	
Myelodysplastic syndrome	3 (6)	3 (6)	
Myelofibrosis	2 (4)	5 (10)	
Other	1 (2)	4 (9)	
Charlson Comorbidity Index	4 (3–4)	4 (3–5)	0.126
Colonization with MDR bacteria, n (%)	5 (11)	3 (6)	0.486
**Admission characteristics**	**Pre-intervention period (n = 56)**	**Intervention period (n = 57)**	***p*** **value**
Reason for admission, n (%)			0.095
Induction	14 (25)	26 (46)	
Consolidation	8 (14)	4 (7)	
Conditioning for autologous HSCT	11 (20)	6 (10)	
Conditioning for allogeneic HSCT	22 (39)	21 (37)	
Microtransplantation	1 (2)	0 (0)	
Duration of neutropenia (days)	14 (10–22)	18 (13–24)	0.121

Data are presented as median (interquartile range) unless otherwise indicated

Colonization with MDR bacteria was defined as colonization with methicillin-resistant *Staphylococcus aureus* or extended-spectrum beta-lactamase producing *Enterobacterales*

*Abbreviations*: HSCT: Hematopoietic stem cell transplantation; MDR: Multidrug-resistant

**Table 2:** Main characteristics of febrile neutropenia episodes

	Pre-intervention period (n = 56)	Intervention period (n = 57)	*p* value
Total number of fever episodes, n	87	103	
Number of fever episodes per admission	1 (1–2)	2 (1–2)	0.08
Number of fever episodes per admission, n (%)			
1	33 (59)	24 (42)	
2	15 (27)	20 (35)	
3	8 (14)	13 (23)	
Type of fever episodes, n (%)			0.35
MDI	19 (22)	32 (31)	
Bacteremia	9 (48)	18 (57)	
Urinary tract infection	4 (21)	3 (9)	
CVC-related infection	4 (21)	8 (25)	
* Clostridioides difficile* infection	1 (5)	0 (0)	
Fungal infection	1 (5)	3 (9)	
CDI	36 (41)	39 (38)	
Oral mucositis/dental	9 (25)	6 (15)	
Abdominal	9 (25)	8 (21)	
Skin and soft tissue	0 (0)	6 (15)	
Pulmonary	7 (20)	8 (21)	
Perianal	4 (11)	3 (8)	
CVC	3 (8)	3 (8)	
Multiple	4 (11)	5 (12)	
FUO	32 (37)	32 (31)	
Duration of fever episode (days)	4 (2–7)	3 (2–5)	0.326
Fever recurrence, n (%)	31 (36)	46 (45)	0.207
Time to fever recurrence (days)	6 (4–10.5)	7 (4–10)	0.896
Sepsis, n (%)	7 (8)	9 (9)	0.864
Septic shock, n (%)	2 (2)	3 (3)	1

Data are presented as median (interquartile range) unless otherwise indicated

*Abbreviations*: CDI: Clinically documented infection; CVC: Central venous catheter; FUO: Fever of unknown origin; MDI: Microbiologically documented infection

### Applicability and compliance with de-escalation and discontinuation strategies

Applicability and compliance with early de-escalation and discontinuation strategies are presented in Table 3. Early de-escalation or discontinuation of EAT was applicable in 56% of FN episodes in the pre-intervention period and 69% of FN episodes in the intervention period. Reasons for non-applicability included presentation with septic shock, failure to achieve the required duration of treatment and apyrexia or failure to obtain clinical recovery before neutrophil recovery. Compliance with early de-escalation and discontinuation strategies was significantly higher in the intervention period than in the pre-intervention period.

**Table 3:** Applicability and compliance with early de-escalation and discontinuation strategies

	Pre-intervention period (n = 87)	Intervention period (n = 103)
Episodes when strategies applicable, n (%)	49 (56)	71 (69)
Compliance*, n (%)	4 (8)	55 (77)
De-escalation	4 (8)	12 (17)
De-escalation followed by discontinuation	0 (0)	6 (8)
Discontinuation	0 (0)	37 (52)
Non-compliance*, n (%)	45 (92)	16 (23)
Lack of timeliness^a^	2 (4)	11 (16)
Continuation	40 (82)	3 (4)
Escalation	3 (6)	2 (3)
Episodes when strategies not applicable, n (%)	38 (44)	32 (31)

*p < 0.001 for the comparison of compliance between pre-intervention and intervention periods

^a^Lack of timeliness was defined as a delay of more than 48 h in the application of early discontinuation or de-escalation strategies

In univariate analysis, compliance with early de-escalation and discontinuation of EAT was more frequent in patients with acute myeloid leukemia (OR 2.62, 95% CI [1.25–5.5], *p* = 0.011*)* and in patients from the intervention period (OR 38.67, 95% CI [12.07–123.9], *p <* 0.001*)*. In multivariate analysis, inclusion in the intervention period was the only factor associated with compliance with early de-escalation and discontinuation strategies (OR 49.27, 95% CI [12.53–193.68], *p <* 0.001*).* The logistic regression model is included in the Supplementary material (Table S2).

### Antibiotic consumption

Antibiotic consumption data are presented in Table 4. In the intervention period, the total number of DOT per 1000 hospital days decreased by 24%. Following the implementation of the intervention, there was a significant decrease in overall antibiotic consumption: overall antibiotic DOT per stay decreased by a median of 8 days while LOT decreased by a median of 6 days. The number of AFD between the onset of the first FN episode and neutrophil recovery was significantly greater in the intervention period. When assessing specific antibacterial agents, carbapenem DOT and anti-resistant Gram-positive agents DOT (e.g., glycopeptides, lipopeptides and oxazolidinones) were significantly lower in the intervention period. The number of days of therapy with piperacillin-tazobactam, cefepime, aminoglycosides and quinolones did not differ significantly between the two periods.

**Table 4:** Antibiotic consumption

	Pre-intervention period (n = 56)	Intervention period (n = 57)	*p* value
DOT/1000 hospital days, n	860	648	
DDD/1000 hospital days, n	1216	946	
Overall antibiotic DOT	28 (20.75–40)	20 (10–32)	**0.006**
LOT	21 (14–24.25)	15 (8–23)	**0.006**
Antibiotic-free days	0 (0–0)	2 (0–8)	**< 0.001**
Aminoglycosides, n (%)	20 (36)	16 (28)	
DOT	1 (1–1)	1 (1–1)	0.553
Carbapenems, n (%)	26 (46)	20 (35)	
DOT	9 (6.25–15.25)	5.5 (4–8)	**0.017**
Piperacillin-tazobactam, n (%)	55 (98)	49 (86)	
DOT	14 (8–21.5)	10 (6–17)	0.084
Cefepime, n (%)	4 (7)	20 (35)	
DOT	6.5 (5–10.5)	7.5 (4–10)	0.953
Piperacillin-tazobactam/Cefepime/Aztreonam, n (%)	56 (100)	56 (98)	
DOT	14.5 (8–22)	12.5 (8–20)	0.61
Quinolones, n (%)	5 (9)	5 (9)	
DOT	3 (2–8)	4 (2–8)	0.733
Anti-resistant Gram-positive agents, n (%)	36 (64)	36 (63)	
DOT	11.5 (7–18)	8 (4–12)	**0.017**

Data are presented as median (interquartile range) unless otherwise indicated

DOT and LOT are expressed in days

Anti-resistant Gram-positive agents include glycopeptides, lipopeptides and oxazolidinones

*Abbreviations*: DDD: Defined daily dose; DOT: Days of therapy; LOT: Length of therapy

### Clinical outcomes

Clinical outcomes are displayed in Table 5. We found no statistical difference in the rates of ICU admission (9% during each period) and 30-day mortality (0% vs. 5%) between the two periods. However, none of the three deaths in the intervention period were related to bacterial complications (i.e., hepatic veno-occlusive disease following allogeneic HSCT, coronavirus pneumonia, invasive pulmonary aspergillosis). Two *Clostridioides difficile* infections were diagnosed during the pre-intervention period and none during the intervention period. Finally, length of stay did not differ significantly between the two periods.

**Table 5:** Clinical outcomes

Outcomes	Pre-intervention period (n = 56)	Intervention period (n = 57)	*p* value
*Clostridioides difficile* infection	2* (4)	0 (0)	0.243
ICU admission	5 (9)	5 (9)	1
Bacterial infection-related ICU admission	2 (4)	2 (4)	1
30-day mortality	0 (0)	3 (5)	0.243
Bacterial infection-related 30-day mortality	0 (0)	0 (0)	1
In-hospital mortality	2 (4)	3 (5)	1
Length of stay (days), median (IQR)	30.5 (27–41)	34 (29–39)	0.308

Data are presented as number (%) unless otherwise indicated

*Abbreviations*: ICU: Intensive care unit; IQR: Interquartile range

*One *Clostridioides difficile* infection occurred after neutrophil recovery and antibiotic discontinuation

## Discussion

These findings suggest that the implementation of an AMS intervention is a safe and effective tool to optimize antibiotic consumption in high-risk neutropenic patients. Implementation of early de-escalation and discontinuation strategies resulted in lower overall antibiotic consumption as well as lower carbapenem and anti-resistant Gram-positive agents consumption. The AMS intervention did not significatively affect aminoglycosides and quinolones consumption, which may likely be explained by the low consumption of these molecules prior to the implementation of this intervention. It should be noted that cefepime use was more frequent during the intervention period due to growing resistance to piperacillin-tazobactam in the department between 2015 and 2020. Despite decreased overall antibiotic consumption, we found no detrimental effects of the AMS intervention on 30-day mortality, ICU admission and length of stay. Assessing clinical outcomes is key to reassure clinicians that AMS interventions do not negatively affect patient safety, in order to increase adherence to these interventions. Patients receiving corticosteroids were excluded from the intervention due to the potential masking effect of corticosteroids on fever, which may have delayed the reintroduction of antibiotics in these patients. Acceptability of early de-escalation or discontinuation of EAT was high following the implementation of the AMS intervention. Indeed, clinician compliance was observed in more than 75% of episodes when strategies were applicable. It is worth mentioning that late adaptation of EAT was the most frequent form of non-compliance in the intervention period whereas continuation of EAT was the most frequent form of non-compliance in the pre-intervention period. High compliance to early adaptation of EAT in this study can likely be attributed to the collaborative and persuasive approach throughout the implementation of this AMS intervention, which was maintained through regular face-to-face meetings and ongoing feedback. Handshake stewardship has been shown to be an effective and sustainable approach to decrease anti-infective use [28]. Exploring the barriers and facilitators to appropriate fever management and antibiotic prescribing by hematologists is key to enhance the adoption of early adaptation of EAT in high-risk neutropenic patients.

In line with previous studies, early de-escalation and discontinuation of EAT was safe in this study. Two prior larger studies found that the implementation of an AMS intervention resulted in improved clinical outcomes, including decreased ICU admission or overall mortality [22, 23]. We believe that our study did not have sufficient power to detect such difference. Given the growing evidence on the safety of early adaptation of EAT, ECIL guidelines should be more widely implemented in hematology departments. In contrast to previous studies [17, 23], we observed a significant decrease in overall antibiotic consumption after implementation of the intervention. This may be explained by the fact that despite lower carbapenem consumption in the intervention phase, there was no increase in the consumption of other beta-lactams, indicating a high uptake of both de-escalation and discontinuation strategies. Previous studies have reported discordant results regarding the occurrence of fever recurrence after early adaptation of EAT [13, 14, 21, 22, 25]. In our study, fever recurred after more than 35% of febrile episodes, without significant variation between the two periods. Moreover, the frequency of microbiological documentation did not differ significantly between the two periods. These results suggest that early adaptation of EAT, as recommended by ECIL guidelines, does not increase the risk of infection relapse. Given the frequency of fever recurrence and its occurrence independent of EAT adaptation, de-escalation and discontinuation strategies should be applied in the same manner after fever recurrence.

One of the strengths of this study is the use of a validated metric to measure antibiotic consumption [27, 29]. Antibiotic consumption was expressed as DOT to account for the use of combination therapy, in order to reflect total antibiotic exposure. Moreover, antibiotic consumption was measured at the level of the study population and not for the whole department and therefore more directly reflects the effects of the AMS intervention. Finally, the required study sample size, based on an expected reduction of overall antibiotic consumption by 25%, was achieved.

This study has several limits, including the retrospective inclusion of patients in the pre-intervention period which may have introduced confounding factors to the analysis, such as the type of chemotherapy used. Evaluation of the impact of the AMS intervention was performed according to a before-after design, which did not account for pre-existing trends unlike interrupted time series (ITS) analysis [30]. However, ITS analysis could not be performed due to insufficient time points during the two periods. Furthermore, the before-after design without a concomitant control group did not account for potential confounding external factors, which may have affected our results. This study could also have benefited from assessing the impact of the AMS intervention on bacterial antibiotic resistance. Indeed, reducing selective pressure on bacterial flora by decreasing unnecessary antibiotic exposure in high-risk neutropenic patients may positively impact local antimicrobial resistance patterns and should be the subject of future research. Moreover, some AMS interventions have been shown to have unsustainable long-term effects after removal of the intervention, despite initial positive effects on antibiotic consumption [31]. Considering that AMS interventions are labor-intensive and cost-intensive, studies on the cost-effectiveness and sustainability of these interventions in high-risk neutropenic patients are needed.

## Conclusion

The implementation of a multifaceted AMS intervention led to high compliance with early de-escalation and discontinuation of EAT by the hematology team. High application of de-escalation and discontinuation of EAT resulted in lower overall antibiotic consumption, without negatively affecting clinical outcomes. These results suggest that AMS interventions, based on multidisciplinary collaboration and personalized clinical recommendations, are a safe and effective tool to optimize the quality of antibiotic prescribing and fever management in high-risk neutropenic patients. Prospective studies are needed to evaluate the sustainability and long-term impact of AMS interventions on antibiotic consumption, clinical outcomes and antibiotic resistance in high-risk hematological patients.

### Electronic supplementary material

Below is the link to the electronic supplementary material.

**Supplementary Material 1:** **File S1.** Febrile neutropenia guidelines. **Figure S1.** Decision algorithm for introduction of empirical antibiotic therapy for febrile neutropenia. **Figure S2.** Decision algorithm for reassessment of empirical antibiotic therapy with piperacillin-tazobactam or cefepime. **Figure S3.** Decision algorithm for reassessment of empirical antibiotic therapy with carbapenem. **Table S1.** Description of bacterial species and antibiotic susceptibility isolated from blood cultures in bacteremia. **Table S2.** Logistic regression model: factors associated with compliance with early de-escalation and discontinuation strategies

## Data Availability

The datasets used and/or analysed during the current study are available from the corresponding author on reasonable request.
