# Supplementary material for: Efficacy of an antimicrobial stewardship intervention for early adaptation of antibiotic therapy in high-risk neutropenic patients
Source: Antimicrob Resist Infect Control. 2024 Jan 17;13:5. doi: 10.1186/s13756-023-01354-5 (PMC10795280; doi:10.1186/s13756-023-01354-5)
Supplement: Supplementary file 1 — Supplementary Material 1: File S1. Febrile neutropenia guidelines. Figure S1. Decision algorithm for introduction of empirical antibiotic therapy for febrile neutropenia. Figure S2. Decision algorithm for reassessment of empirical antibiotic therapy with piperacillin-tazobactam or cefepime. Figure S3. Decision algorithm for reassessment of empirical antibiotic therapy with carbapenem. Table S1. Description of bacterial species and antibiotic susceptibility isolated from blood cultures in bacteremia. Table S2. Logistic regression model: factors associated with compliance with early de-escalation and discontinuation strategies [file 13756_2023_1354_MOESM1_ESM.docx]

## **Supplementary material 1**

**File S1:** Febrile neutropenia guidelines

Pre-intervention and intervention periods

In line with ECIL guidelines, EAT with a beta-lactam was started immediately after onset of fever in neutropenic patients, after blood cultures were taken. During both periods, carbapenem use was restricted to patients with prior colonization or infection with extended-spectrum $\beta$-lactamase-producing bacteria and in patients with septic shock. A single injection of aminoglycosides was administered in patients with sepsis or septic shock. An agent active against resistant Gram-positive bacteria (GPB) (e.g., glycopeptide, lipopeptide or oxazolidinone) was added, similarly during both periods, in patients with suspected catheter-related infection, skin and soft tissue infection or severe mucositis and in patients with septic shock.

Intervention period only

EAT was systematically reassessed between 48 and 72 hours after introduction.

- *De-escalation strategy*

If EAT with carbapenem was started based on prior colonization or infection with resistant pathogens and no microbiological documentation was available, de-escalation was encouraged in stable patients who had been afebrile for at least 48 hours. If an agent active against GPB was started based on focal clinical signs and no GPB was documented, its stopping was encouraged in stable patients upon resolution of focal clinical signs. If carbapenem and anti-resistant GPB therapy were started in the context of septic shock and the patient stabilized on treatment without documented infection, no particular change was encouraged.

- *Discontinuation strategy*

Without clinical nor microbiological documentation, EAT discontinuation was encouraged after 72 hours in stable patients who had been afebrile for at least 48 hours. In clinically or microbiologically documented infections, discontinuation of appropriate antibiotic therapy was encouraged after 7 days and after infection was microbiologically eradicated, clinical resolution was obtained and fever had resolved for at least 4 days.

- *Approach to persistent or recurrent fever*

If fever persisted in stable patients without new clinical signs, changes in antibiotic therapy were discouraged and the diagnostic work-up was continued, including repeated blood cultures and computed tomographic of chest and sinuses. If fever recurred after antibiotic discontinuation, EAT was immediately reintroduced, after new blood cultures were taken.

**Figure S1:** Decision algorithm for introduction of empirical antibiotic therapy for febrile neutropenia

Abbreviations: AMS: Antimicrobial stewardship; CVC: Central venous catheter; ESBL: Extended-spectrum beta-lactamase; Pipe-tazo: piperacillin-tazobactam; qSOFA: Quick SOFA; SST: Skin and soft tissue

**Figure S2:** Decision algorithm for reassessment of empirical antibiotic therapy with piperacillin-tazobactam or cefepime

Abbreviations: AMS: Antimicrobial stewardship; CVC: Central venous catheter; EAT: Empirical antibiotic therapy; GPB: Gram-positive bacteria; Pipe-tazo: piperacillin-tazobactam; SST: Skin and soft tissue

**Figure S3:** Decision algorithm for reassessment of empirical antibiotic therapy with carbapenem

Abbreviations: AMS: Antimicrobial stewardship; CVC: Central venous catheter; EAT: Empirical antibiotic therapy; GPB: Gram-positive bacteria; Pipe-tazo: piperacillin-tazobactam; SST: Skin and soft tissue

**Table S1:** Description of bacterial species and antibiotic susceptibility isolated from blood cultures in bacteremia

|  | **Pre-intervention period** | **Intervention period** |
| --- | --- | --- |
| Number of bacteremia episodes | 9 | 18 |
| Gram-positive species, n (%) | 4 (44) | 2 (11) |
| *Staphylococcus aureus* | 1 | 0 |
| Susceptible to methicillin | 1 | N/A |
| *Streptococcus viridans* | 2 | 1 |
| *Enterococcus faecalis* | 0 | 1 |
| *Enterococcus faecium* | 1 | 0 |
| Gram-negative species, n (%) | 5 (56) | 16 (89) |
| *Enterobacterales* species | 5 | 11 |
| *Escherichia coli* | 4 | 4 |
| *Klebsiella pneumoniae* | 0 | 6 |
| *Enterobacter cloacae* | 0 | 1 |
| Multiple *Enterobacterales* species | 1 | 0 |
| Susceptible to piperacillin-tazobactam | 3 | 5 |
| Susceptible to cefepime | 5 | 10 |
| Susceptible to imipenem | 5 | 11 |
| Extended-spectrum beta-lactamase producer | 0 | 1 |
| *Pseudomonas species* | 0 | 3 |
| *Pseudomonas aeruginosa* | N/A | 2 |
| *Pseudomonas* non-aeruginosa species | N/A | 1 |
| Susceptible to piperacillin-tazobactam | N/A | 3 |
| Susceptible to cefepime | N/A | 3 |
| Susceptible to imipenem | N/A | 3 |
| Anaerobic species | 0 | 2 |

**Table S2:** Logistic regression model: factors associated with compliance with early de-escalation and discontinuation strategies

| **Variables** | **Univariate** | | **Multivariate** | |
| --- | --- | --- | --- | --- |
|  | **OR (95% CI)** | ***p* value** | **OR (95% CI)** | ***p* value** |
| Age $<$65 years | 1.02 (0.48–2.18) | 0.951 |  |  |
| Charlson Comorbidity Index |  |  |  |  |
| $\quad\leq$ 2 | 0.38 (0.12–1.15) | 0.087 | 1.74 (0.3–10.23) | 0.541 |
| $\quad>$ 2 | Reference |  | Reference |  |
| Hematologic disease |  |  |  |  |
| AML | 2.62 (1.25–5.5) | **0.011** | 0.81 (0.26–2.53) | 0.715 |
| Other | Reference |  | Reference |  |
| Type of chemotherapy |  |  |  |  |
| Induction | 1.22 (0.59–2.5) | 0.592 |  |  |
| Other | Reference |  | Reference |  |
| Typer of fever episode |  |  |  |  |
| FUO | 1.13 (0.55–2.34) | 0.733 |  |  |
| Other | Reference |  | Reference |  |
| Duration of febrile episode (days) |  |  |  |  |
| $\quad\leq$ 3 | 1.26 (0.6–2.66) | 0.536 |  |  |
| $>$3 | Reference |  | Reference |  |
| Period |  |  |  |  |
| Intervention | 38.67 (12.07–123.9) | **< 0.001** | 49.27 (12.53–193.68) | **< 0.001** |
| Pre-intervention | Reference |  | Reference |  |

Abbreviations: AML: Acute myeloid leukemia; CI: Confidence interval; FUO: Fever of unknown origin; OR: Odds-ratio
